# Biomechanical Features of Graphene-Augmented Inorganic Nanofibrous Scaffolds and Their Physical Interaction with Viruses

**DOI:** 10.3390/ma14010164

**Published:** 2020-12-31

**Authors:** Michael Gasik, Roman Ivanov, Jekaterina Kazantseva, Yevgen Bilotsky, Irina Hussainova

**Affiliations:** 1 School of Chemical Engineering, Aalto University Foundation, FIN-00076 Aalto, Finland; 2 Department of Mechanical and Industrial Engineering, Tallinn University of Technology, EE-19086 Tallinn, Estonia; roman.ivanov@taltech.ee (R.I.); irina.hussainova@taltech.ee (I.H.); 3 Center of Food and Fermentation Technologies, EE-12618 Tallinn, Estonia; jekaterina.kazantseva@tftak.eu; 4 Seqvera Ltd. Oy, Helsinki University Central Hospital Area, FIN-00290 Helsinki, Finland; seqvera.research@gmail.com

**Keywords:** nanofibers, alumina, graphene, virions, biomechanics, adherence, anisotropy

## Abstract

Nanofibrous substrates and scaffolds are widely being studied as matrices for 3D cell cultures, and disease models as well as for analytics and diagnostic purposes. These scaffolds usually comprise randomly oriented fibers. Much less common are nanofibrous scaffolds made of stiff inorganic materials such as alumina. Well-aligned matrices are a promising tool for evaluation of behavior of biological objects affected by micro/nano-topologies as well as anisotropy. In this work, for the first time, we report a joint analysis of biomechanical properties of new ultra-anisotropic, self-aligned ceramic nanofibers augmented with two modifications of graphene shells (GAIN scaffolds) and their interaction of three different viral types (influenza virus A, picornavirus (human parechovirus) and potato virus). It was discovered that nano-topology and structure of the graphene layers have a significant implication on mechanical properties of GAIN scaffolds resulting in non-linear behavior. It was demonstrated that the viral adhesion to GAIN scaffolds is likely to be guided by physical cues in dependence on mutual steric factors, as the scaffolds lack common cell membrane proteins and receptors which viruses usually deploy for transfection. The study may have implications for selective viral adsorption, infected cells analysis, and potentially opening new tools for anti-viral drugs development.

## 1. Introduction

Nano-biomaterials are being extensively studied on their interactions with cells and biologic environment. They already have a well-documented ability to stimulate and trigger certain cells receptors and, therefore, affect cells behavior and their fate [1,2]. Furthermore, their additional surface functionalization with nano-species to be used in ongoing research and different medical devices. Carbon in different forms has a wide application in biology and medical sciences, and with regards to nano-structure, one of the most used forms is carbon nano-tubes (CNTs), which is essentially composed of rolled graphene sheets [3]. Graphene, as an intrinsically a 2D nanomaterial, can contribute or even serve as a stimulus for many attention-grabbing biological interactions, depending on the geometry, morphology and nano-structure of the substrates [1,2,3].

The authors have recently demonstrated applications of unique scaffolds, composed of self-aligned graphene-augmented inorganic (alumina) nano-fibers (GAIN). Ultra-high anisotropy (over 10^6^:1) and porosity (over 90%) of fibers were shown to result in thrilling effects over the cell cultures seeded; including but not limited by the suppression of inflammatory markers in human mesenchymal stem cells (hMSC) and peripheral mononuclear blood cells [4], the neurogenic-type differentiation of hMSC without any specific differentiation media [5], and the different gene expression and the reactivity of various types of cancer cells [6]. These findings allowed to formulate a phenomenon of auto-mechanoinduction in the cells, where ultra-high mechanical anisotropy of the substrate acting on two different levels (from 10–20 nm to cm scale) has triggered unusual (mutually confusing) reactions of the cells and, as a result, unexpected gene expression and cells differentiation [7].

This phenomenon was explained by interaction of cells membrane proteins (and eventually focal adhesions) with every single fiber in one direction, whereas in orthogonal direction, all adhesions were facing the same fiber. The fiber distance (15~30 nm) allows rather flexible relative displacement of the fibers and focal adhesions of the cells which were attached to these fibers. In this way the cells “feel” the scaffold stiffness in the direction perpendicular to the fibers in a completely differently way (as few kPa only), then in another–parallel-direction (~300–400 GPa). The stiffness, which is various in different directions, causes confusion in cells mechano-sensing response and results in the features reported in [4,5,6,7]. In this respect, it is of great importance to reveal biomechanical macroscopic properties of the scaffolds at the conditions close to the physiological-relevant ranges [8,9].

Whereas biomechanical and mechanobiological interactions of cells and bacteria with various biomaterials and their surfaces has been extensively studied [10,11,12,13], much less is known about the interactions between biomaterials and viruses. Viruses have lesser dimensions as compared to cells or bacteria, and they are lacking features, which are typical for the cell membrane. Capsid of enveloped viruses made up of the proteins and phospholipids acquired by the virus within the cytoplasm of the cell during the virus extrusion or when it passes from the nucleus to cytoplasm [14,15,16].

The deep understanding of virions interaction with a solid and biological matter is of a paramount importance for proper monitoring, control, diagnostics, and treatment of diseases in plants, animals, and humans. Viruses are infectious particles that consist of a nucleic acid genome and its protective shell, which is composed of proteins and, sometimes, lipids [14]. With a limited capacity to self-replication, viruses require the living cells to infect them in a parasitic way. They can infect essentially all organisms, from bacteria to plants and mammals, and cause a variety of diseases in multicellular organisms [14,15,16]. The genome of enveloped viruses is packed in a protein capsid, which is surrounded by a lipid membrane derived from the host cell. Viruses must simultaneously form a capsid that is stable enough to encapsulate a self-repulsive polyelectrolyte cargo, protect that cargo in many different environments, and remain dynamic enough to deliver that cargo with a minimal genome [16,17].

The routes of viral adhesion, binding and intro-cellular penetration are paid by a great deal of attention, but a physical adsorption or an adherence of viruses to scaffolds, especially to the new nanofibrous scaffolds, is unknown. Although such adherence seemingly does not affect the virus activity, it is of concern for the analysis of the viral interactions with the cells and cultures on such scaffolds [14,16], for new areas such as virus-incorporated biomimetic nanocomposites for tissue regeneration [18], as well as for the general understanding of complex interaction mechanisms. Moreover, little is known on how enveloped and non-enveloped viruses differ in their adhesion ability to nanofibers. In this sense, it is of a rising significance to understand whether enveloped and non-enveloped viruses are reacting (adhering) to the GAIN scaffolds as the mechanisms and the driving force of adhesion are likely differ from those of cells.

In this work, we have studied interaction of GAIN with different virus types to get answers onto the questions: (1) how much is the difference in biomechanical properties with variation of graphene layers on highly anisotropic alumina nano-fibers, (2) if there is a difference between enveloped and non-enveloped viruses adherence to GAIN, (3) if the steric factor (geometry and size) of the virus plays a role in this process, (4) if the envelopes (in the case of enveloped viruses) affect the adherence to GAIN similarly as was found for the cells, and (5) what are the intrinsic biomechanical properties of enveloped virions to be considered for analysis of possible virions adherence to such materials.

## 2. Materials and Methods

### 2.1. Graphene-Augmentation on Alumina Nanofibers

A ceramic nanofibers network, produced by a recently developed process of controlled liquid phase oxidation of an aluminum melt [19], was chosen as a substrate for carbon deposition. The network of these nanofibers represents a meso-porous complex structure consisting of aligned self-assembled nanofibers with a single nanofiber diameter ranged 5–50 nm and a narrow distribution of nanofibers diameters throughout one block. The specific surface area (with Brunauer–Emmett–Teller (BET) method) was found to be in the range of 140–175 m^2^/g depending on fibers diameter and morphology. The as-produced fibers are mostly composed of partially hydrated (2–6 wt.%) γ-alumina phase that can be converted into α-alumina by heat treatment at temperatures exceeding 1250 °C [20]. Transmission electron microscopy (TEM) and scanning electron microscopy (SEM) studies show that a single alumina nanofiber may have variations in diameter of approximately 0.5–1.5 nm, demonstrating sometimes twinning or local breakage. 

Differential scanning calorimetry (DSC) performed earlier (data in [20]) has been combined with high resolution HR-TEM observations of specific zig-zag surface structural features. Those studies have revealed possible unsaturated Al^3+^ sites linked to three O atoms. The cation Al^3+^ binds to hydroxyl OH^−^, and protons form H-bonds localized on the neighboring Al-O-Al bridge resulting in formation of both terminal OH-groups and bridging groups indicated by the IR spectra shown in [20]. Therefore, the nanofibers in this structure are hold together mainly by weak hydrogen forces that can easily be broken in liquid solutions. Due to this, original scaffolds are not directly suitable for biological studies without treatment such as by augmentation with graphene.

A few-layered highly defected graphene sheets were deposited onto the bundle of aligned γ-alumina nanofibers of 40 ± 3 nm in diameter and about 5 cm in length exploiting a single-step chemical vapor deposition (CVD) approach. This original laboratory installation includes a four-channel gas system with input pressure gauges (Swagelok Co., Solon, OH, USA), digital flow controllers (ALICAT Scientific, Inc., Tucson, AZ, USA) for two channels (methane and hydrogen for purging and removal of residual oxygen), manual low flow metering valves for two other channels (nitrogen and argon as purge and protective gases) and one common flow-meter. The main part—a processing reactor—is made of quartz tube with a length of 150 mm, an inner diameter of 10 mm, and a wall thickness of 1.5 mm placed in specially designed tube furnace. The mass flow-meter allows measurement of up to 5 L/min and mass flow controllers—up to 0.5 L/min for H_2_ and up to 0.2 L/min for CH_4_. Methane of 99.5 vol.% purity (UN 1971, 2.5) and technical nitrogen 99.5 vol.% (UN 1066) have been used in these experiments. The catalyst-free CVD process was performed at atmospheric pressure and temperature of 1000 °C in methane (CH_4_, flow of 50 cm^3^/min) and nitrogen (N_2_, flowing of 500 cm^3^/min) gas stream [21,22]. Time of the reaction was adjusted depending on the desired structure of the coating; for example, mass gain of 15% of the substrate weight was reached during 20 min of processing. 

**Figure 1:**
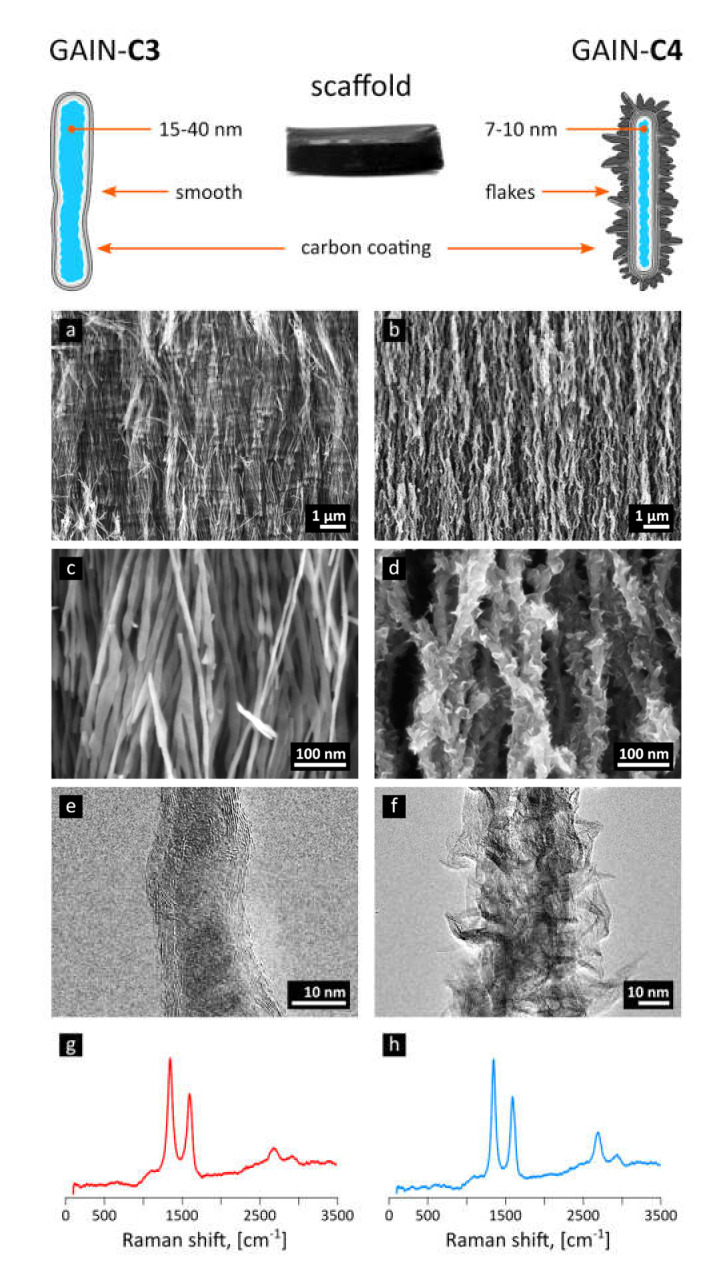
The schematic representation of synthesized GAIN scaffolds and their structure from SEM and TEM images for the type “C3” (**a**,**c**,**e**) and “C4” (**b**,**d**,**f**), together with their respective Raman spectra (**g**,**h**).

The carbon produced through decomposition of methane in the presence of nitrogen envelops (augments) the oxide nano-fibers, forming graphene-like shells along the fiber. The morphology of carbon−alumina hybrid nanostructures is controlled by the deposition time, ratio between carbon source (methane) and carrier gas (nitrogen), and the flow rate [21,23]. The deposition time of 20 min (mass gain of 15%) corresponding to 3–7 layers of graphene has led to type of scaffold marked here as “C3”), Figure 1a,c,e, and deposition time of 120 min produced structures decorated with graphene-like flakes or protrusions, scaffold type marked here as “C4” [22], Figure 1b,d,f. These images were obtained with TEM (JEOL JEM-2200FS HR-TEM with two C_s_-correctors, 200 kV field emission gun (FEG) and in-column energy filter (Omega Filter) configured to produce a high-end energy filtered imaging) and SEM (Zeiss HR Gemini FESEM Ultra 55 equipped with Bruker EDX system ESPRIT 1.8 suitable for energy-dispersive X-ray measurements).

Raman spectroscopy, used for characterization of carbon layers, was performed with the help of Horiba Jobin Yvon HR800 high resolution Raman spectrometer equipped with a green Nd: YAG laser (532.1 nm), a red He-Ne laser (632.8 nm), and a multichannel CCD detection system in the backscattering configuration.

### 2.2. Biomechanical Properties

GAIN scaffolds biomechanical characterization was performed as referred to § 10 of Annex I of EU Medical Devices Regulations (2017/745) to align the data with the physiologically important limits (frequency 1 Hz, deformation amplitudes for the typical cells size range) [8,10,24]. Specimens geometry was controlled with ±1 µm precision using non-contact laser micrometer (Metralight, Inc., Burlingame, CA, USA). 

Mechanical tests were performed with DMA242E “Arthemis” (Netzsch Gerätebau GmbH, Selb/Bayern, Germany) with ± 0.5 nm displacement resolution, automatic pre-conditioning, and baseline subtraction. All materials were stepwise loaded from 10 to 50 µm amplitude at 1 Hz in air (“dry”) and in phosphate-buffered saline (PBS) solution (“wet”), with the loading cycles repeated 10 times for every specimen. Dynamic loading was deformation-controlled in compression. The specimen’s fibers orientation perpendicular to the loading direction. When the amplitude is rising, also dynamic force is increased proportionally to the apparent stiffness of the sample to be determined. The static part of the force was set to be 0.1% of the dynamic force to ensure a contact of the sample with the sample holder and the probe is preserved through the experiments. Data analysis was performed with the integrated DMA software “Proteus” (Netzsch Gerätebau GmbH, Selb/Bayern, Germany) and with a model-free approach using idempotent post-processing known as BEST (Biomaterials Enhanced Simulation Testing; Seqvera Ltd., Helsinki, Finland), described in more detail in [25,26].

### 2.3. Viruses and Their Preparation

**Figure 2:**
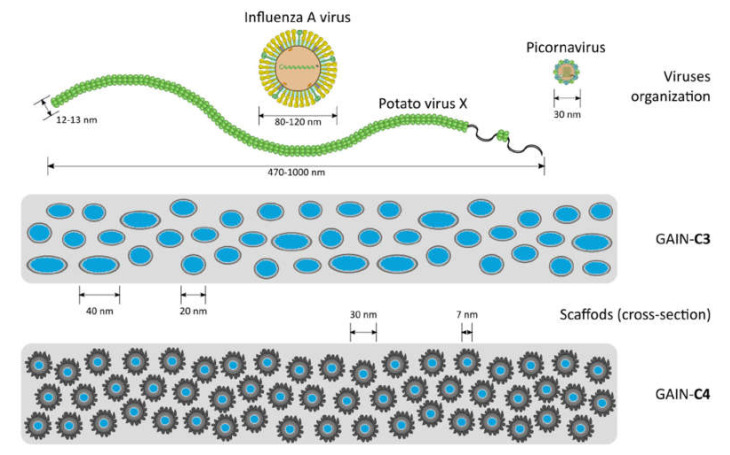
Schematic representation of virus organization vs. the scale of the GAIN scaffolds structure (the fibers are arranged perpendicularly to the view).

Three different virus types were used in this work (Table 1). The dimensions of these viruses to GAIN scaffolds are schematically shown in Figure 2. It is seen that sterically these viruses are substantially different so it is expected they will interact with the GAIN scaffolds also in a different way.

Alphaphlexiviruses, including known plant pathogens such as potato virus X (PVX), are helically symmetrical non-enveloped viruses [14]. PVX is known as a potential platform for biomedical applications as a model to understand virus-host interactions and due to diverse agricultural and biomedical applications of PVX-based vectors [14,27,28]. This virus was chosen as a model for initial experiments of PVX binding to C3 and C4 scaffolds. PVX was propagated in Nicotiana tabacum plants and lysate of PVX-infected plant cells were prepared at 2 days post-appearance of mosaic patterns on the leaves that indicate productive infection. Lysate of non-infected cells was used as control in all experiments. PVX does not tolerate freezing well, thus fresh cell lysate has to be prepared for each experiment. Lysates were pre-cleared by centrifugation at 8000 g for 30 min at 4 °C and the supernatant was used for further studies.

Enveloped virus IAV (influenza A virus) causes annual epidemics of respiratory disease in humans, as well as recently spreading pandemic CoVID-19 coronavirus. IAV are one of the major causative pathogens of human acute respiratory disease responsible for seasonal epidemics and reoccurring pandemics of influenza, which poses a significant threat to human health and economic development [29]. IAV also causes symptomatic disease in birds and thus imposes threat to poultry farming (“bird flu”). There are no effective measures to control IAV; therefore, this virus continuously requires attention from the scientific community, surveillance agencies, public and private parties. Here IAV (strain A/WSN/33) was propagated at a BSL-2 containment facility in Madin-Darby Canine Kidney cells in virus-growth medium (VGM; Dulbecco’s Modified Eagle’s Medium (DMEM) supplied with 1% glutamate, 0.2% of bovine serum albumin (BSA), 1 µg/mL tosyl phenylalanyl chloromethyl ketone-treated trypsin (TPCK-trypsin) and 1% antibiotic and antimycotic solution (ABAM)) for 48–72 h. After the cytopathic effect (CPE) was close to 100%, the supernatant containing the virus was collected and pre-cleared by centrifugation at 15,000g for 15 min. The supernatant containing the virus was aliquoted and stored at −70 °C.

Non-enveloped viruses consist of just a genome and icosahedrally or helically symmetrical protein capsid. One of the smallest non-enveloped viruses are picornaviruses with icosahedral symmetry, which can cause a variety of diseases in humans [15,30]. Human parechovirus (HPeV) is a non-enveloped RNA virus of Picornaviridae family with at least 17 identified genotypes, of which types 1 and 3 are the most common [31]. HPeV infection is often asymptomatic or associated with a mild respiratory or gastrointestinal disease in children. Human parechovirus type 3 (HPeV3) can cause a severe sepsis-like illness in young infants (aged < 3 months), including central nervous system (CNS) infection and may be associated with a long-term neurodevelopmental delay later in childhood [30]. The recombinant HPeV3 continued to show a remarkable stability in its capsid amino acid sequence, indicating a clear need for development of a vaccine or immunotherapeutics to reduce the severity of HPeV3 [15,30,31]. HPeV1 virus is extremely common worldwide; >99% of individuals have experienced HPeV1 infection by the age of two and it rarely causes a severe disease, but is commonly used in HPeVs research, as it is highly similar to HPeV3, but is much easier to handle in the laboratory conditions [15].

Here HPeV1 (strain Harris) was propagated at a BSL-2 containment facility in HT29 cells in McCoy’s medium supplied with 1% glutamine, 2% fetal bovine serum (FBS), and 1% ABAM for 72–96 h. After CPE was close to 100%, the dead cells were collected together with supernatant. The solution was frozen and thawed 3 times and then pre-cleared by centrifugation at 15,000 g for 15 min. The supernatant comprising the virus was aliquoted and stored at −70 °C.

### 2.4. Virus Binding to GAIN Scaffolds

For PVX virus, the scaffolds (n = 12) were pre-incubated in binding buffer (0.01 M phosphate buffer, pH = 7.5). No pre-incubation was done when binding to dry scaffolds was tested. Lysate binding was done at room temperature for 2 h, after which scaffolds were washed 3 × 5 min with binding buffer. For IAV and HPeV1 viruses the scaffolds (n = 6 per scaffold and virus type) were pre-incubated in virus infection medium for 1 h. Virus binding to scaffolds was done at room temperature for 1 h following fixing the scaffolds with 4% paraformaldehyde (PFA) and used for immunofluorescence analysis.

Immunoblotting for detection of PVX used horseradish peroxidase-conjugated primary anti-PVX C antibody produced in mouse and diluted 1:500 in phosphate-buffered saline (PBS). Immunoblot was visualized by chemiluminescence using a standard protocol. A specific band that corresponding to PVX C protein size (34 kDa) was observed in the lysate of infected, but not of non-infected leaves (data not shown), so PVX was confirmed to be present in infected plant lysate and it can be specifically detected using anti-C antibody. The plant lysate bound to scaffolds was fixed with 4% PFA for 10 min at room temperature, following immunofluorescence (secondary anti-mouse antibody conjugated to Alexa488 fluorophore was used diluted 1:5000 in PBS with 0.1% Tween 20). For detection of IAV primary anti-H1N1 IAV glycoprotein polyclonal antibodies (antibody 1 and antibody 2) produced in rabbits were used. For HPeV1, primary polyclonal anti-HPeV antibody was used also generated in rabbits. Antibody dilution was 1:100 in all cases, and the same visualization method as for PVX above (with secondary anti-mouse antibody conjugated to Alexa488 fluorophore) was used. Scaffolds were visualized by light microscopy.

The fluorescence images were semi-quantitatively analyzed with the ImageJ software [32]. Briefly, every image was calibrated first with the scale bar to ensure the same area is being processed. Color threshold operation was performed on all images by limiting major hue component histogram (maximizing green component), setting lower lightness limit at maximal intensity, and limiting saturation to the band where the intensity was detectable. An alternative method was also tested by stretching brightness histogram first, following the lowest contrast limit setting to the maximal intensity. Both methods were giving similar results in enhanced image quality. After this, a green component was converted into a binary mask and area of the image (%) covered by this mask was calculated. The procedure was repeated three times from the original images to get averaged values of the coverage vs. control. For scanning electron microscopy (SEM), the scaffolds were dried at 60 °C, coated with platinum and visualized in a Zeiss ULTRA-55 SEM at 30,000–250,000 × magnification.

### 2.5. Plaque Assay for IAV

Plaque assay was used to quantify IAV in solution before and after binding to the scaffold. Briefly, MDCK cells were plated in 6-well microplates and infected with series of 10-fold dilutions of virus stock before and after scaffold binding to scaffolds. For each infection 200 µL of virus dilution was used. The infection was done in VGM. The cells were incubated for 1 h at 37 °C rocked every 15 min to prevent drying and subsequently overlaid with 1% Avicel solution in minimum essential medium (MEM) supplied with 1% glutamate, 1% ABAM, 0.2% BSA and 1µg/mL TPCK-trypsin. After 48 h incubation, the cells were washed twice with PBS, fixed with 4% PFA for 30 min and stained with 0.2% crystal violet in 25% methanol. Virus titre was calculated in plaque-forming units (pfu) per mL as follows:

*Virus titre (pfu/mL) = number of plaques × dilution factor × 5.* (1)

To estimate the efficiency of virus binding to scaffold, the virus titre after binding was compared to the virus titre before binding.

### 2.6. Statistical Analysis

The presence of leverage points was checked with hat matrix diagonal components and those points which did not fit Stephen’s rule were removed. The presence of outliers was made by calculating Cook’s distances, and those data points exceeding unity value were removed from analysis. The consistency of regression was independently checked by application of Theil-Shen estimator and the goodness of fit normality and significance by Nelson-improved Anderson-Darling test. Heteroscedacity of residuals was estimated with RUNS test and the residuals autocorrelation by Durbin-Watson parameter.

## 3. Results and Discussion

### 3.1. Properties of GAIN Scaffolds

**Figure 3:**
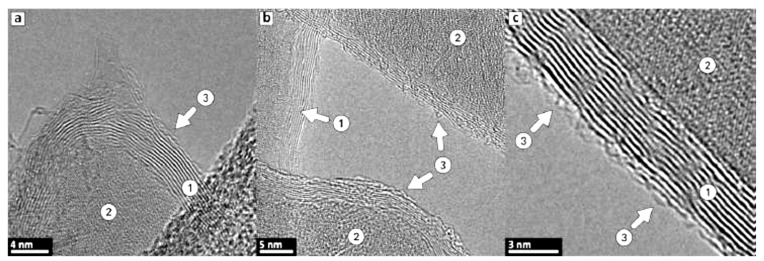
TEM images of graphene coatings around the alumina nanofiber (pictures (**a**–**c**) represent different locations). Label (1) points to stacked carbon layers, (2) marks a substrate nanofiber, and (3) shows outer highly defective layer.

The main characteristics of the Raman spectra of produced C3 and C4 structures [21,23] are disorder-induced (D) and well-recognized (G) peaks at around 1350 and 1590 cm^−1^, respectively (Figure 1g,h). A single symmetric peak at 2680 cm^−1^ indicates a few-layered graphene, as there is no splitting into an asymmetric doublet typical for graphite; moreover, the relatively narrow bands suggest nanocrystalline structure of layers. Generally speaking, the Raman modes are resembling the features representative for multi-walled carbon nanotubes (MWCNT); indeed, the graphene augmented inorganic nanotubes (GAIN) are essentially the graphene sheets wrapped around the ceramic core, Figure 1. TEM images of 7–10 graphene layers developed on the sample (gained 17% of the weight) are demonstrated in Figure 3.

**Figure 4:**
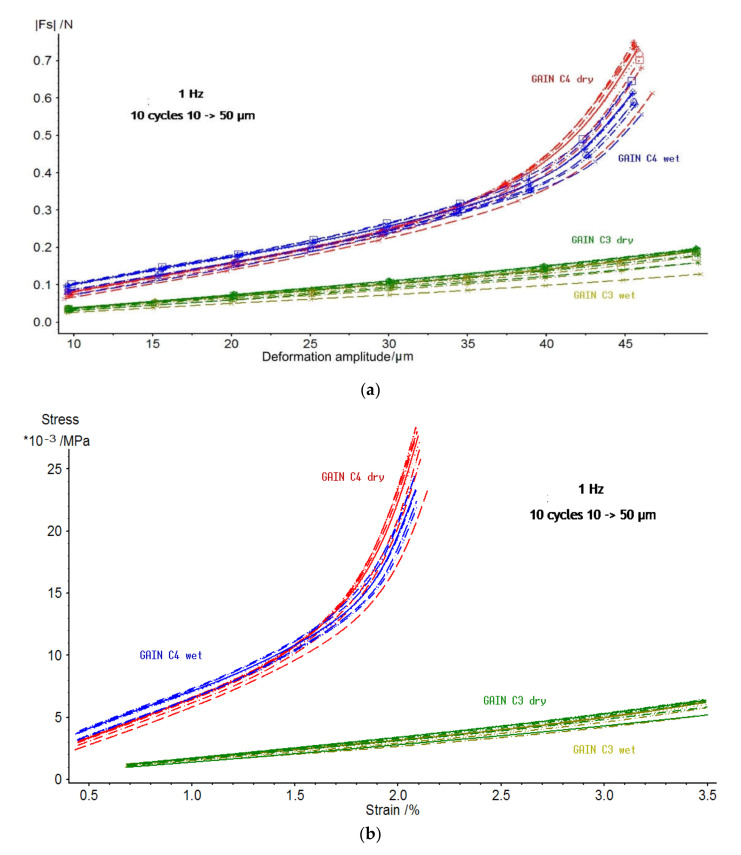
(**a**) Dynamic force (N) vs. dynamic deformation (µm) for C3 and C4 GAIN scaffolds in dry and wet (immersed conditions; (**b**) data of figure (**a**) converted into compressive stress and true strain expressed in % to the starting thickness of the specimen.

Mechanical properties of GAIN scaffolds were assessed for static and dynamics components. For the static component of the force and displacement, only the minor and statistically insignificant changes and differences between the scaffolds C3 and C4 were detected. The GAIN scaffolds are substantially hydrophilic, however, even in the case of fluid absorption or movement at macro level, the scaffolds did not affect static shrinkage not swelling (would have been seen as “negative” stiffness). The data for dependence of dynamic force amplitude (N) vs. deformation amplitude (µm) are shown in Figure 4 (every specimen was loaded and tested 10 times). There is a substantial difference in behavior between C3 and C4 materials, but not so much between dry (in air) and wet (in the fluid media) cases. No hysteresis between the loading cycles was observed.

The data of Figure 4a were normalized by converting a force into a stress and deformation into a strain (as specimens had different dimensions). The stress-strain curves for 1 Hz and for 10 cycles of loading are presented in Figure 4b indicating a remarkable difference between C3 and C4. The C3 substrate undergoes nearly linear behavior in all studied range of strains; nonetheless, the material C4 (either dry or wet state) deviates from the linearity already after 1.5% of strain.

Therefore, it is possible to conclude that augmented graphene morphology (Figure 1) determines the differences in mechanical qualities of the GAIN scaffolds rather than the conditions (dry or wet) under which these scaffolds were tested. As may be seen from Figure 1, C4 graphene shell has significantly higher nano-roughness than smoother C3 type. One can assume that mutual adherence of the GAIN fibers is therefore stronger for C4. Additionally, smooth C3 surface is likely to mover easier relatively to each other, although in this study single fibers nano-mechanical behavior was not studied. Despite of the reason for this mechanical behavior, this means that a credible extraction of tangential (slope) modulus, especially for C4, is impossible with a classical Hooke’s relation of stress to strain (it is mathematically possible at the very low strains, but this value does not have much practical importance to the whole deformation range).

Due to the non-linear behavior of the materials, a model-free idempotent analysis [25,33,34,35] has been applied comprising the time-convolution of the stress input lined to the observed deformation. Experimental stress and strain data are always some functions *F(x,t)* of time and spatial coordinates, and these functions have their respective Laplace transforms. Hence, there should be a general mathematical solution [25] with the convolution integral [33,34]. It is known that convolution integrals do not in general have a closed analytical form, however, they can be obtained as such for the simple loading (stimulation) patterns, for example, creep, linear ramp, or harmonic case [25]. For the latter, authors [10,25,26] have previously shown that the dynamic stress/strain ratio (“dynamic stiffness”) can be expressed as:

(2) $$\frac{\sigma_{dyn}}{\varepsilon_{dyn}}=C_{\omega 0}\times (\alpha \times (\omega ))\times \omega^{\alpha (\omega )}$$

where *σ_dyn_* is the applied dynamic stress amplitude, *ω*—Circular frequency, *C_ω0_ = E_0_ × (τ_0_×ω)^α^* is the viscostiffness (quasi-property in units of kPa·s^α^) [25], *α*—Dynamic material memory parameter, *E_0_*—Intrinsic dynamic elasticity, *τ_0_*—Intrinsic characteristic time. This Equation (2) time-convolutes the specimen loading history at every frequency without Fourier transform or assumptions of a material model (Maxwell, Burger, standard linear solid, Prony series, etc.), nor local differentiation [25].

**Figure 5:**
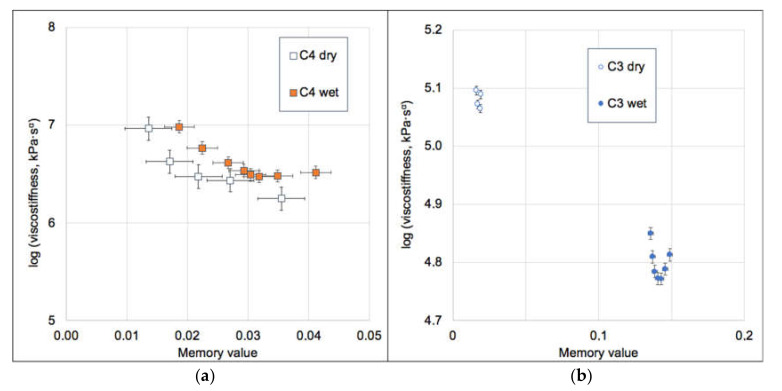
Dependence of the GAIN viscostiffness at 1 Hz vs. their memory values for C4 (**a**) and C3 (**b**). Error bars represent standard error. Note significant differences of memory value between wet and dry C3 scaffolds, but very close values for C4.

The values of “material memory” *α* must be positive to ensure causality principle (no response of any system is observed before the stimulus has been applied). Low *α* values indicate that material has short memory which is less affected by the loading history of the material, and high *α* values indicate that the previous deformations have a longer effect in time. The values of *E_0_* and *τ_0_* do not depend on time or frequency, but the memory value (*α*) usually depends on frequency and might depend also on deformation (strain). These dependences bring major non-linearity in the materials behavior. The variation of *C_ω0_* vs. material memory (*α*), Figure 5 exhibits the obvious non-linearity reflecting the behavior demonstrated in Figure 4. Using the method described in [25,26], it is possible to extract values of the invariant modulus *E_0_* for the specimens under consideration.

For both wet and dry conditions, the invariant modulus of C4 is almost 10-fold higher than for C3 (Table 2). The numerical values of the dependence of the memory value on the true logarithmic strain (ε) are included in Table 2 as the constitutive equations, which can be used for prediction of the bending stiffness of the GAIN substrates at 1 Hz and up to 50 μm of deformation. The mechanically less stiff C3 scaffolds exhibit a substantial increase in the memory value (from ~0.02 to ~0.14) upon wetting, which indicates a considerable role of the fluid phase in mechanical energy dissipation. On the contrary, the stiffer C4 scaffolds are practically unaffected by the presence of fluid and persist their dynamic properties. These differences are considered mostly affecting stem cells and cancer cells behavior via auto-mechanoinduction phenomenon discovered recently [7]. The meaning of the invariants (2) relating the value of *E_0_* can be interpreted as the purely elastic contribution to the dynamic stress/strain amplitudes ratio (“the true stiffness”), and *(τ_0_ω)^α^* —as a viscous correction to the stiffness, vanishing at the purely static loading cases [25].

The smaller the values of *τ_0_* and *α* are, the less dynamic stress/strain ratio (stiffness) would depend on frequency. Here *E_0_* and *τ_0_* do not depend on frequency, and they are not complex values, but the true elasticity and the (model-free) characteristic time of the material (which might be seen as a characteristic time for that material Deborah number). It is noteworthy that a simple decomposition to elastic and viscous parts is not possible (alike in simple spring-dashpot models of linear viscoelasticity) because of the non-linearity of the system (scaling and superposition properties do not hold in general for a non-linear system). It is also notable that these elastic and viscous contributions cannot be simply related to a storage (real) and a loss (imaginary) parts calculated in linear viscoelasticity as obtained via Fourier transform, since the presence of a material memory (even when not being frequency-dependent) makes the product *E_0_* *× (τ_0_* *× ω)^α^* being non-linear.

### 3.2. Virus Interactions with GAIN Scaffolds

**Figure 6:**
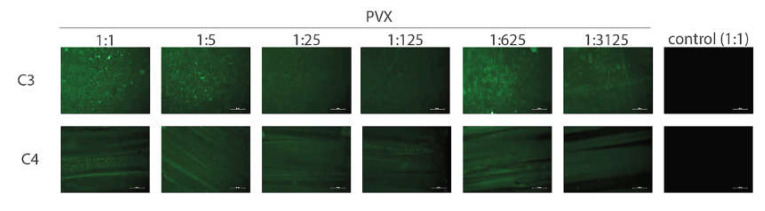
Detection of potato virus X (PVX) on C3 and C4 scaffolds using immunofluorescence. The tested dilutions of infected plant lysate are indicated above the figure (control = non-infected lysate). Color corresponds to presence of PVX C and reflects binding to scaffold. Bar = 50 µm.

The GAIN C3 and C4 scaffolds were pre-incubated in a binding buffer for 2 h and incubated at room temperature with the lysate of infected and non-infected leaves for 2 h. PVX binding to both C3 and C4 scaffolds was clearly detected (Figure 6); no respective signal was detected in the non-infected control. Dilution of infected cell lysate up to even 3125-fold did not prevent detection of the virus, indicating that the PVX amount in the infected cell lysate is likely high. Consequently, both C3 and C4 scaffolds may be confirmed to bind the PVX effectively. Attempts to visualize the PVX on the C3 and C4 scaffolds using SEM were not successful (data not shown), likely due to the PVX instability when subjected to SEM conditions (PVX would be degraded during e-beam irradiation).

Next tests were performed with the non-treated GAIN scaffolds to find whether the PVX can bind the dry scaffold without any pre-treatment with the binding buffer. The fraction of the infected cell lysate was applied to C3 scaffold and immunofluorescence was done immediately for the PVX detection. The C3 scaffold, which was pre-treated with the binding buffer and incubated with the infected cell lysate, served as a positive control for binding. Non-infected cell lysate was used as a negative control. It was observed that the PVX successfully bound to both pre-treated and dry C3 scaffolds (data not shown). This result indicates that no specific treatment of the GAIN scaffolds is required for PVX binding.

**Figure 7:**
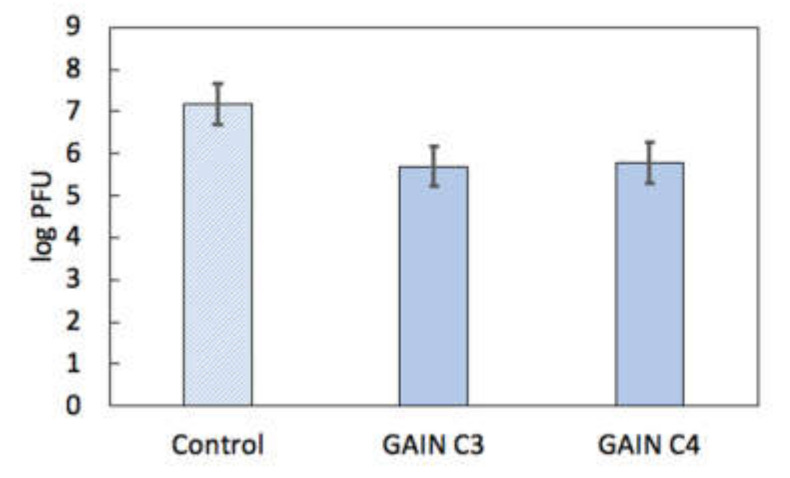
HPeV1 adherence results for GAIN scaffolds (log plaque-forming units (PFU) scale). Error bars show standard error.

First tests of the HPeV1 binding shown no clear specific fluorescent signal neither on C3 nor C4 scaffolds. Possible explanations for this were that (1) there is little or no HPeV1 binding to scaffolds; or (2) the fluorescence signal could not be detected due to technical limitations. To resolve this issue, a quantitative analysis of HPeV1 binding to GAIN scaffolds was performed. GAIN C3 and C4 scaffolds (n = 4, ~2 × 2 × 3 mm each type) were incubated for with 10^7^ pfu of HPeV1 in total volume of 1 mL. The titre of HPeV1 after incubation (1) with scaffolds was quantified using plaque assay and compared to the titre of HPeV1 (1.5 × 10^7^ pfu/mL) that was not incubated with the scaffolds. HPeV1 titre dropped down to 5 × 10^5^ pfu/mL after incubation with C3 scaffolds and down to 6 × 10^5^ pfu/mL with C4 scaffolds (original images with the data are available from the authors), i.e., in 25–30 times. This indicates that both C3 and C4 have capacity to bind HPeV1, without major differences between the capacity of these scaffolds, Figure 7.

Binding of IAV to scaffolds was assessed quantitatively in a similar way. Approximately 5 × 10^5^ pfu of IAV were incubated with GAIN C3 and C4 scaffolds (n = 3, size ~2 × 2 × 3 mm) for 1.5 h at room temperature. Virus titre post incubation was determined using plaque assay and the virus stock not incubated with scaffolds was used as a control. The IAV titre before incubation with the scaffolds was 5 × 10^5^ pfu/mL, IAV titre after incubation with C3 scaffolds was 8 × 10^5^ pfu/mL, and IAV titre after incubation with C4 scaffolds was also 5 × 10^5^ pfu/mL. This result showed no apparent binding of IAV to the GAIN scaffolds, unlike in the HPeV1 case.

**Figure 8:**
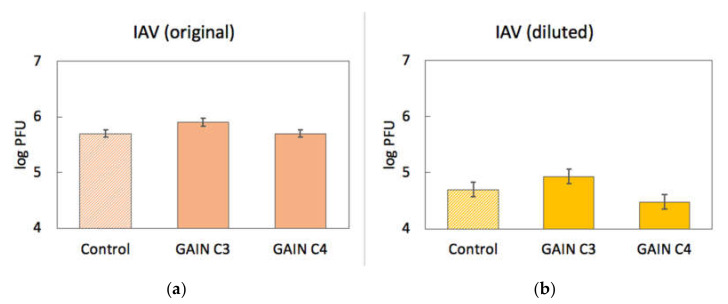
Results of IAV (influenza A virus) binding to GAIN scaffolds (log PFU scale) with original (**a**) and diluted (**b**) virus titres. Error bars show standard error.

To check if IAV binding to GAIN scaffolds would be beyond detection limits of experiments, 1 mL of diluted IAV stock (5 × 10^4^ pfu/mL) was again incubated with C3 and C4 GAIN scaffolds (n = 6), and the IAV titre was quantitated before and after incubation as described above. Virus titre before incubation with the scaffolds was 5 × 10^4^ pfu/mL. Virus titre after incubation with C3 scaffolds was 8.5 × 10^4^ pfu/mL and after incubation with C4 scaffolds was 3 × 10^4^ pfu/mL. This result also has indicated no apparent drop of IAV titre after incubation with C3 and C4 scaffolds, supporting previous observation that IAV does not seem to interact with GAIN scaffolds (Figure 8).

The results (Table 3) show that PVX from freshly prepared infected plant lysate binds well to both C3 and C4 scaffolds. Pre-treatment of GAIN scaffolds is therefore not required for an effective binding of PVX. For HPeV1 and IAV binding to C3 and C4 scaffolds it was found that both scaffolds could bind HPeV1, but neither of these scaffolds could efficiently bind IAV. It might be observed that non-enveloped viruses tested (PVX, HPeV1) can adhere to GAIN scaffolds of different morphology (Figure 2), but an enveloped virus (IAV) cannot adhere despite that the envelope structure and composition is somewhat closer to cellular membranes [4,14,15,29]. Therefore, besides an effect of envelope presence, the steric factor might be more significant in adherence capacity to GAIN scaffolds. The equivalent diameter of PVX is about 15–20 nm and it geometrically nearly perfectly fits into the inter-fibrillar space of GAIN (20–40 nm), whether or not their augmented graphene has different morphology. The extra feature for PVX is that it could also potentially adhere along the nanofibers, which increases possibilities for larger adsorption capacity.

Picornaviruses (and HPeV1 in particular) due to their small sizes (~30 nm) are being closer to potentially available binding positions. For enveloped IAV (>90–100 nm), this would be however difficult, as a virus envelope is not so flexible as it would be in the case of the cell membranes [5,6,14].

### 3.3. Biomechanics of IAV and Its Adherence

It was reported [36] that influenza viral envelope is about 10 times softer than IAV protein-capsid coat, but stiffer than a liposome, with these differences being due to contribution of membrane-associated proteins. Most viral capsids were estimated to have their elastic modulus of order of 1–10 MPa, whereas for IAV it was assessed to be about 1 GPa [36,37,38]. The burst strain of most non-enveloped capsids was marked to be ~10%, so significant deformations associated with virus adherence to a stiff substrate are not a favorable factor.

**Figure 9:**
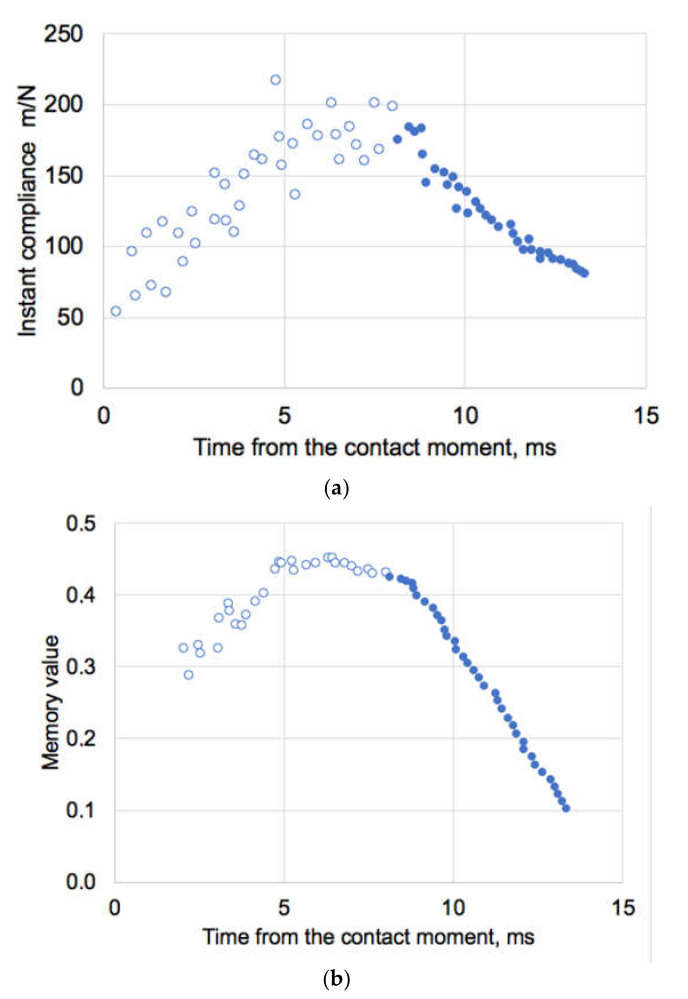
(**a**) Instant mechanical compliance (m/N) of IAV by AFM recalculated from data [36] and (**b**) respective memory values. Open symbols are for Phase I and closed for Phase II deformation as presented in [36]. Maximal compliance values are in this case corresponding to maximal memory values.

We have applied the idempotent method [25] to evaluate data [36] of AFM indentation of IAV A/X31 strain, which have reported envelope stiffness of ~0.02 N/m (equal to compliance 1/0.02 = 50 m/N) at indentation rate of 2 µm/s. The force measured in [36] was clearly seen of having two different branches with different slopes which were considered to represent different stages (phases I and II) of the indentation process. From these experimental AFM data there is indeed changes in the compliance (maximal ~200 m/N) which occur about 8 ms after the contact of the indenter has been established with the viral envelope. This maximal compliance corresponds to the minimal stiffness of the system, Figure 9a. During dynamic loading and with a clear non-linear response, traditional Hookean analysis is unlikely to provide feasible results. Hence, by numerical integration of the data [36], we have obtained a time-convoluted memory values (Figure 9b) and respective viscostiffness as function of every time step point. This have been made without any assumptions of the linearity of the indenter-viral envelope system.

By convoluting the data [36] for the case of contract rate deformation, the constitutive equation for deformation of influenza virus would take a form:

(3) $$S(t)=\frac{F(t)}{z(t)}=\frac{S_{0}}{\Gamma (2-\alpha (t))}{\left(\frac{\tau_{0}}{t}\right)}^{\alpha (t)}$$

where *S(t)*—a reverse of the compliance—is the stiffness observed at time t, *z(t)* is the displacement in nm, *F(t)* is the applied force in nN, *S_0_* is the invariant stiffness, α(t) is the memory value (Figure 9b), *τ_0_* is the characteristic time, and Γ(·) is the gamma-function. The viscostiffness product *S_0_τ_0_^α^* has a clear exponential dependence on memory α, if the process comprises a single phase:

(4) $$\ln\left(S_{0}\tau_{0}^{\alpha }\right)=\ln S_{0}+\alpha (t)\cdot \ln\tau_{0}$$

**Figure 10:**
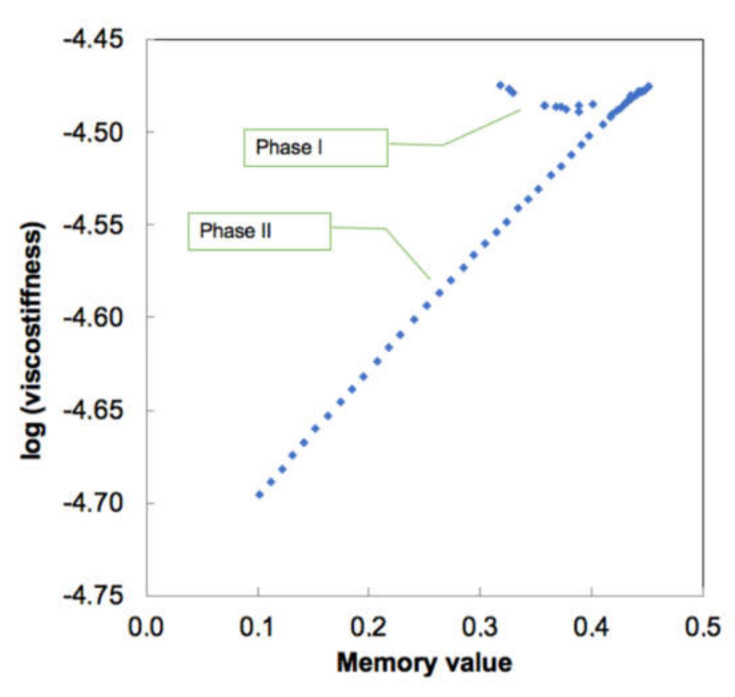
Extracted values of viscostiffness (N·s^α^/m) and memory values for influenza virus deformation [35]. Note a linear dependence for Phase II and nearly constant value for Phase I.

As seen from Figure 10, this only holds for the deformation Phase II, and for Phase I (despite changes in α), data are just scattered. We may hypothesize that Phase I, as was also assumed by authors [36], is not a “true” deformation of the virus envelope but rather a combination of the local events of spikes bending, deformation and cross-interactions between them and the indenter [37]. Hence the stiffness of 0.02 N/m obtained by a tangent slope (= Hooke’s law) is not correct, as there is actually a spectrum of different stiffnesses which cannot be individually quantified. In Figure 10 this is reflected by practical independence of the viscostiffness product ln (*S_0_ × τ_0_^α^*) from α, which directly implies for the unity of characteristic time for Phase I and gives *S_0_* = 0.0113 N/m for Phase I and 0.0086 N/m for Phase II. The latter value can be considered as an intrinsic real stiffness of the influenza viral envelope. Characteristic reactivity time for Phase II deformation is 1.9 ms—this is the minimum required time (at the conditions in [36]) for the viral envelope to deform properly without being affected by history of the loading.

In respect to the subject of the present study, the most relevant are the Phase I data in Figure 9 and Figure 10. GAIN nanofibers might be considered as “indenters” but in the present experiments they do not have an explicit loading on the viruses, as the latter settle onto the fibers by gravity, capillary, and other surface forces. The invariant stiffness of the GAIN scaffolds (Figure 9) is about 10^5^ times higher than the *S_0_* value for IAV at Phase I, so both C3 and C4 can be considered as rigid materials vs. IAV structures. Any extra attraction forces, which are acting on the virus-scaffold system must therefore overcome about 10–20 nm deformation (~0.1–0.2 nN force) to let the virion approach closely to GAIN fibers. As was seen from the experiments, this however does not happen whether or not GAIN fibers have smooth (C3) or more rugged (C4) structure, whereas the latter might be thought to have more potential anchoring sites for IAV spikes.

It is reasonable to assume that large, enveloped viruses (arbo-, myxo-, paramyxo-, pox- and herpes-families) would unlikely adhere to the GAIN scaffolds, whereas small, non-enveloped (picorna-, reo-, papo-, adeno-viridae) viruses might stick on more easily—They do not need similar mechanical deformation, even if their capside is more rigid than an envelope [38]. An interesting yet open feature remains in details of adherence of coronaviridae, as their spike receptors [39] might be anchoring to GAIN nanofiber sites easier than for the IAV case. Based on this study, it is possible to suggest that viruses binding to GAIN-scaffolds is significantly affected by physical cues, directed by geometry (steric) factors, presence of need of an extra mechanical deformation, and surface interactions (such as via electrostatic charge), rather than by mainly biological or biochemical ones (as in the case of viral-cells interactions [4,5,6,7]).

It also reasonable to assume that the effect of automechanoinduction [7] seen on GAIN with different cells, does not play a role for viruses as they are unable to replicate without a host cell, do not have the same processes of proliferation, growth and differentiation as for stem cells, neither (in the case of large enveloped viruses) are strain-compliant to ultra-anisotropic GAIN substrates. However, this does not exclude possibility that virus-infected cells, subjected to auto-mechanoinduction on GAIN scaffolds, might lead to interesting unexpected effects—viruses would in such case interact with different intracellular signals and factors not commonly observed on flat 2D or randomized 3D substrates.

The study performed was preliminary, and has several limitations, such as small power size and limited number of tests carried out. It was not also possible yet to draw conclusions about the kinetics of PVX binding to the scaffolds. HPeV1 adsorption per surface unit of scaffold is needed to be further estimated in a separate set of experiments where scaffolds of controlled size will be incubated with HPeV1 and virus titre would be quantified by plaque assays in multiple replicates in more detail. Similarly, binding kinetics would require an additional set of experiments.

## 4. Conclusions

In this work, for the first time, graphene augmented inorganic nanofiber (GAIN) scaffolds were used to discover potential adherence (binding) of different virus types. GAIN scaffolds were shown to represent a novel tool for directing and influencing cells behavior without biological or pharmacological cues, but there was no knowledge about the interactions of virions with these nanomaterials.

Plant potato virus (PVX) and two human pathogenic viruses (HPeV1 and IAV) binding was analyzed, and the results indicate that smaller non-enveloped viruses to have more binding capacity than larger enveloped ones. It is possible to assume enveloped viruses have unfavorable geometry (steric factor) and their envelopes are not able to accommodate to GAIN ultra-anisotropic nano-structure, unlike cells membranes.

This study has a three-fold implication. First, aligned ultra-long nanofibers alike GAIN might be an effective substrate to capture small non-enveloped viruses without pre-incubation, and perhaps even to separate them from enveloped ones, if both are present in the lysate. Second, if the viruses binding is indeed more a physical process, GAIN-like systems might be beneficial in studying peculiarities of virions interaction with different matter to understand their physical and nano-mechanical properties for development of better viral control and anti-viral products. Third, this approach might be also extended to studies of infected stem cells or cancer cells as a complementary tool for understanding transfection and development of new antiviral and anticancer therapies.

## Figures and Tables

**Table 1:** Viruses used in this work [14,15].

Type	Characteristic	Envelope	Dimensions
Potato virus X (PVX)	Plant pathogen	No	500–1000 × 10–15 nm, helical rods
Influenza A virus (IAV)	*Orthomyxoviridae*; human pathogen; ether sensitive	Yes	80–120 nm, helical capsid
Human parechovirus (HPeV)	*Picornaviridae*; neonatal pathogen; non-sensitive to ether	No	20–30 nm, cubic capsid

**Table 2:** The invariant parameters and constitutive equation for GAIN scaffolds (<50 μm deformation).

Condition	Dynamic Stress/Strain Ratio, kPa	
	C3 Scaffold	C4 Scaffold
Dry	176.39 × (6.38 × 10^−3^ × *ω*)^0.1455ε + 0.0152^	1570.30 × (3.67 × 10^−16^ × *ω*) ^−1.2663ε + 0.0397^
Wet	163.40 × (1.22 × 10^−1^× *ω*)^−0.6330ε + 0.1546^	1695.60 × (5.18 × 10^−13^ × *ω*) ^−1.4018ε + 0.0497^

**Table 3:** Summary of the virus binding to GAIN scaffolds.

Virus Type	Scaffold Pretreatment	Adherence to	
		C3 Scaffold	C4 Scaffold
PVX	Does not affect	Very good	Very good
HPeV1	-	Good	Good
IAV	-	No	No

## Data Availability

Original data and figures, including plaque test images and binding test pictures not shown in the paper, are available from the corresponding authors.
